# Datasets of *in vitro* clonogenic assays showing low dose hyper-radiosensitivity and induced radioresistance

**DOI:** 10.1038/s41597-022-01653-3

**Published:** 2022-09-08

**Authors:** Szabolcs Polgár, Paul N. Schofield, Balázs G. Madas

**Affiliations:** 1 Doctoral School of Physics, ELTE Eötvös Loránd University, Budapest, Hungary; 2 Environmental Physics Department, Centre for Energy Research, Budapest, Hungary; 3 Department of Physiology, Development and Neuroscience, University of Cambridge, Cambridge, United Kingdom; 4 Department of Physical Chemistry and Materials Science, Budapest University of Technology and Economics, Budapest, Hungary

**Keywords:** Data mining, Data publication and archiving, Data acquisition, Databases

## Abstract

Low dose hyper-radiosensitivity and induced radioresistance are primarily observed in surviving fractions of cell populations exposed to ionizing radiation, plotted as the function of absorbed dose. Several biophysical models have been developed to quantitatively describe these phenomena. However, there is a lack of raw, openly available experimental data to support the development and validation of quantitative models. The aim of this study was to set up a database of experimental data from the public literature. Using Google Scholar search, 46 publications with 101 datasets on the dose-dependence of surviving fractions, with clear evidence of low dose hyper-radiosensitivity, were identified. Surviving fractions, their uncertainties, and the corresponding absorbed doses were digitized from graphs of the publications. The characteristics of the cell line and the irradiation were also recorded, along with the parameters of the linear-quadratic model and/or the induced repair model if they were provided. The database is available in STORE^DB^, and can be used for meta-analysis, for comparison with new experiments, and for development and validation of biophysical models.

## Abstract (summary)

**Table:** 

Measurement(s)	surviving fraction of cells
Technology Type(s)	optical microscopy
Factor Type(s)	absorbed dose
Sample Characteristic - Organism	Homo sapiens • Chinese hamster • Rattus sp.
Sample Characteristic - Environment	cell culture

## Background & Summary

Clonogenic assay or colony formation assay is an *in vitro* cell survival assay based on the ability of a single cell to grow into a colony; a colony is defined as having at least 50 cells^1^. The surviving fraction (*SF*) of cells as the function of absorbed dose can generally be described by the linear-quadratic ﻿(LQ) model^2^ (Eq. 1). In this model, the fraction of surviving cells decreases exponentially as the function of dose, and this exponential function consists of a linear and a quadratic term. As surviving fraction is normalized to the unirradiated control, it equals 100% at 0 Gy by definition:

1 $$SF={e}^{-\alpha D-\beta {D}^{2}},$$

where *D* is the absorbed dose (Gy) and *α* and *β* are the linear and quadratic parameters describing the radiosensitivity of the cells.

For certain cell lines, however, the surviving fraction at low doses significantly differs from the LQ model^3^. These cell lines exhibit hyper-radiosensitivity (HRS) at very low radiation doses (~0.1 Gy) which is not predicted by extrapolating the cell survival response from higher doses using the LQ model. As the dose increases above ~0.3 Gy, there is an increased radioresistance (IRR) to doses beyond ~1 Gy, where radioresistance is maximal, and cell survival starts to follow the LQ model. As HRS and IRR may have implications for cancer therapy, several biophysical models^4–8^ have been developed, aiming to provide a deeper understanding of the phenomena.

The development and validation of such biophysical models requires raw experimental data, and cell survival data are a key resource in understanding the factors underlying the phenomena of biosensitivity to low dose radiation. Despite improvements in the requirement for authors to make raw data supporting publications publicly available, there is still a significant gap between expectation and delivery^9–11^. Moreover, it is also clear that relying on authors to provide data personally on request is not reliable, and accessibility decreases with time from the data of publication^12^. We have addressed this problem by extracting primary data from published graphics in papers, a strategy not so far attempted at scale, and provide that data in a public database together with a demonstration of the power of data integration and reanalysis, supporting key aims of FAIR data which include interoperability and reuse^13^. Reproducibility of published studies is of increasing concern^14,15^ and we demonstrate here how reproducibility can be assessed using data harvested from prior studies.

Friedrich *et al*.^16^ established a database, the Particle Irradiation Data Ensemble (PIDE), of cell survival experiments published in the literature. Raw data have been added more recently^17^. The focus of their data mining was to support the study of relative biological effectiveness (RBE) for clonogenic cell survival as endpoint, and to provide a benchmark for RBE-predicting models against experimental data. Therefore, only those *in vitro* cell survival experiments are included in PIDE, where data are available on both photon and ion irradiation, excluding important studies of HRS and IRR.

**Fig. 1:**
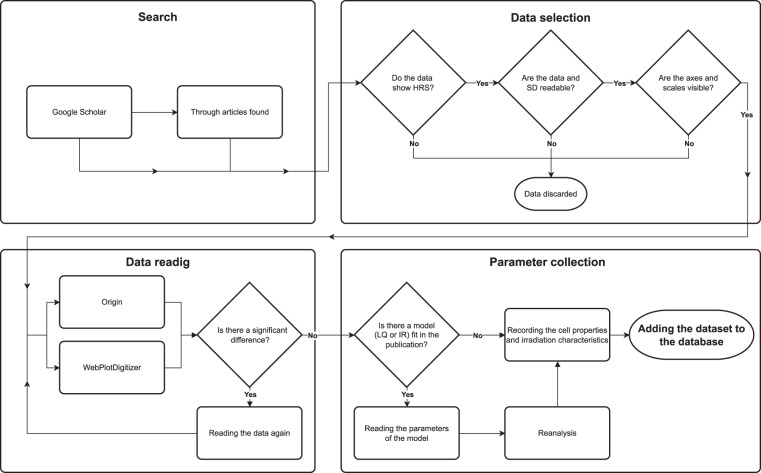
The flow chart describing the steps we used to acquire the datasets for the database.

The aims of the present study were to collect datasets featuring experiments with various cell cultures showing HRS and IRR from published articles in a reproducible and technically sound way and make them publicly available according to the FAIR guidelines^13^. Besides raw data on cell survival and absorbed dose, parameters of the most frequently fitted models, the LQ model and the induced repair model (IR model) were also collected. A schematic overview of the study is provided in Fig. 1.

## Methods

A literature review was performed using the search tool of Google Scholar (https://scholar.google.com/) with the keywords of “low-dose hyper-radiosensitivity”, “low-dose hrs”, and “induced radioresistance”. The references in the articles found were also searched for graphs. The last search was performed on 2^nd^ August 2021. Criteria for a graph to be processed were the following:

(i) a low-dose HRS region could clearly be identified in the graph,
(ii) the data points of the surviving fractions and their uncertainties were readable from the graphs, and
(iii) the axes and the scale of the graphs were clearly visible.

Applying this procedure, 46 articles were found containing 101 datasets^3,18–62^. The oldest articles were published in 1993, while the most recent ones in 2021, so the datasets were taken from a time span of over 25 years. There were a wide variety of cell lines investigated, and different radiation types and dose rates were applied. Some publications were found with graphs which met criterion (i) but not criterion (ii)^63–66^.

Since the last search was performed, other publications were found which could have been included in the database^67–70^. It shows that our search did not find all relevant publications. The database can later be extended with data from these publications.

For each article, the title, the authors, the figure number which the dataset was obtained from, the name of the irradiated cell line, the type of the radiation and its properties (which were characteristic and provided, e.g., dose rate, energy, tube voltage, linear energy transfer) were recorded. If the authors fitted the LQ or the IR model to their data, then those parameters and their standard errors or confidence intervals were also noted, depending on which one was given.

In order to obtain numerical values of surviving fractions, corresponding absorbed doses, and uncertainties of the surviving fractions from the graphs, the applications WebPlotDigitizer4.2 (GNU Affero General Public License v3.0, https://automeris.io/WebPlotDigitizer/) and OriginPro2018 (OriginLab Corporation, https://www.originlab.com/) were used. First, the x and y axes had to be defined with the scale (linear or logarithmic) and by defining two points known for each to determine the size of one unit. After that, numerical data for surviving fractions and the corresponding absorbed doses could be read from the individual data points. Uncertainties of the surviving fractions were determined by reading the minimum and maximum values of the whiskers of each data points. As there is no unique established way of reporting errors in cell survival values^16^, uncertainty of surviving fraction may mean standard deviation or standard error of the mean, and in some cases it is not even mentioned which one was used. For the LQ and IR model fits, the parameters are presented either with standard errors or confidence intervals depending on the preference of the authors. While these two could be calculated from each other, the required information for this is frequently not presented in the article.

To validate the numerical value of the LQ and IR model fits in the articles, a reanalysis was performed on the actual datasets. The LQ model fit was given by the original articles in a total of 24 cases and the IR model fit was given in a total of 59 cases, the results of the reanalysis were compared to the published data. Our fit was considered to be different from the original one if the difference between values of any IR parameters (*α*_*r*_, *α*_*s*_, *β*, and *D*_*c*_) was larger than the sum of their uncertainties. The Levenberg–Marquardt method^71,72^ and the Orthogonal Distance Regression^73^ were used for fitting in the application of OriginPro2018 (OriginLab Corporation, https://www.originlab.com/).

In the LQ model, there are two parameters (*α* and *β*). As the LQ model does not take into account low dose HRS, Eq. (1) was fitted first only to data points above 1 Gy or to the three data points at the highest doses even if any of them were lower than 1 Gy. If this initial fit did not result in the parameters given in the articles, the Eq. (1) was fitted to the entire dataset including the HRS region.

In the IR model^37,74^, the relationship between surviving fraction and absorbed dose can be described by Eq. (2):

2 $$SF={e}^{-{\alpha }_{r}\left(1+\left(\frac{{\alpha }_{s}}{{\alpha }_{r}}-1\right){e}^{-\frac{D}{{D}_{c}}}\right)D-\beta {D}^{2}}.$$

**Fig. 2:**
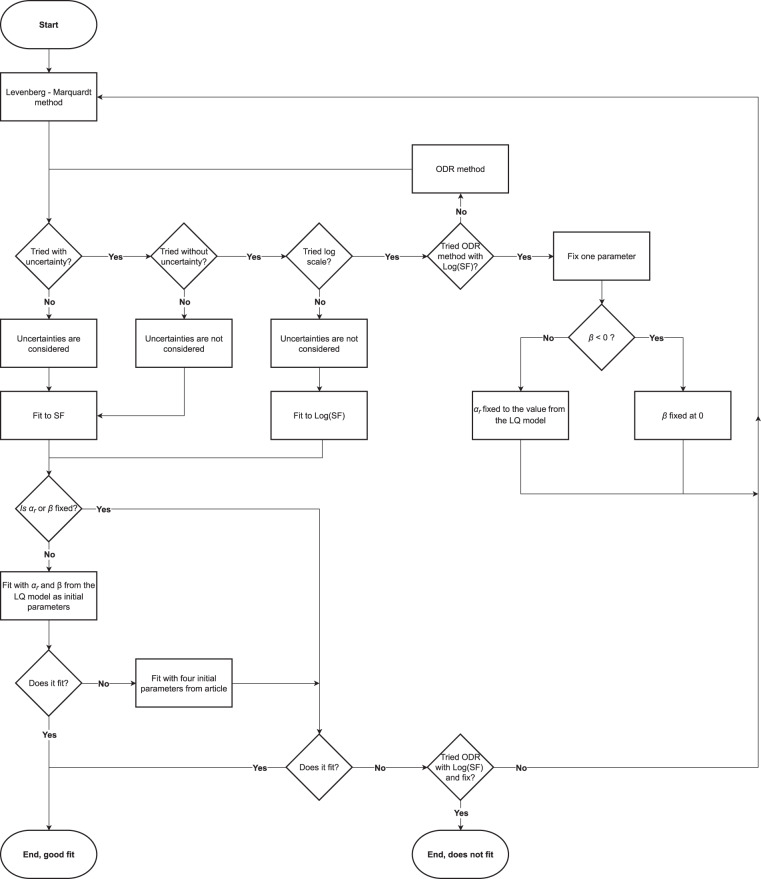
The flow chart describing the fitting method we used to reproduce the﻿ IR model parameters of the original articles.

Here, *β* is the same as in the LQ model, while *α* of the LQ model is replaced by *α*_*r*_ for high doses, and *α*_*s*_ for low doses. *D*_*c*_ is the critical dose or the “transition point” between low-dose hyper-radiosensitivity and induced radioresistance (i.e., when *α*_*s*_ to *α*_*r*_ is 63% complete). As there are four parameters, convergence of the fitting is sensitive to the initial values of the parameters. In order to test whether a fitting method can be found which reproduces the parameters given in the articles, the following protocol was applied, which is also shown in Fig. 2. If one step failed to reproduce the original parameters, the next one was applied.

1. The initial values of *α*_*r*_ and *β* parameters were determined by fitting the LQ model to the surviving fractions measured at absorbed doses higher than 1 Gy, or to the three data points at the highest doses even if any of them were lower than 1 Gy. The initial values of *α*_*s*_ and *D*_*c*_ were set to 1 Gy^−1^ and 1 Gy, respectively. Equation (2) was fit with these four initial values to surviving fractions considering their uncertainty.
2. The initial values of the four parameters were set equal to the parameters in the original publications. Equation (2) was fitted to surviving fractions considering their uncertainty.
3. The initial values were the same as in 1). Equation (2) was fitted to surviving fractions without considering their uncertainty.
4. The initial values were the same as in 2). Equation (2) was fitted to surviving fractions without considering their uncertainty.
5. The initial values were the same as in 1). The logarithm of Eq. (2) was fitted to the logarithm of the surviving fractions without considering their uncertainty.
6. The initial values were the same as in 2). The logarithm of Eq. (2) was fitted to the logarithm of the surviving fractions without considering their uncertainty.
7. Instead of the Levenberg – Marquardt algorithm, the Orthogonal Distance Regression method was applied. The six previous steps were tested until one reproduced the original parameters.
8. The seven previous steps were tested until one reproduced the original parameters with one parameter fixed, and the others fitted. The motivation behind this step is that it is easier to find an optimum with fewer parameters fitted simultaneously.
  - ﻿If the β parameter was negative from the LQ fit, then it was fixed to 0 and the others were fitted.
  - Otherwise, the α_r_ parameter was fixed to the value α of the LQ model fit, and the other parameters were fitted.

## Data Records

The first and second versions of the database have been uploaded to the STORE^DB^ database (https://www.storedb.org/store_v3/index.jsp), which is a repository for data and links to resources of the international radiobiology community, and maintained by the Federal Office of Radiation Protection, Germany^75^. It ensures long-term persistence and preservation of datasets, provides deposited datasets with Digital Object Identifiers, standardised metadata^76,77^, allows access to data without unnecessary restrictions, and provides a licence on each dataset landing page.

The current (second) version (STOREDB:DATASET1252) of the database^78^ contains 101 datasets from 46 articles^3,18–62^ in Microsoft Excel 2016 format (Microsoft Corporation, https://www.microsoft.com/en-gb/microsoft-365/excel). It is publicly available under Creative Commons Attribution license. One dataset contains the surviving fraction of the cell culture (column C) at a given dose in Gy (column B) and the minimum (column D) and maximum value (column E) of the whiskers of the uncertainty of the surviving fraction. The parameters of the fitted function are also recorded if they were given in the original article, either parameters of the LQ model or the IR model or both. The fit type is given in column G. From column H to column X, the different parameters (columns H, L, P, Q, U) are given with their standard errors (columns I, M, R, V) or confidence limits (columns J, K, N, O, S, T, W, X). In column H, *α* refers to the LQ fit, while *α*_*r*_ to the IR fit. If there are parameters or values which were not given in the articles (or no fits were made), then it is indicated with an ‘X’ symbol. If the parameters has no meaning for the given fit (for example the LQ model has only two parameters, *α* and *β*, so the others are not applicable), a ‘-’ symbol is used. Lastly, the cell type (the name of cell line, the species, the organ, the cancer type if applicable) in column Z and the characteristics of the irradiation in column AA are recorded (radiation type, dose rate, energy, tube voltage, linear energy transfer, etc.).

## Technical Validation

The technical quality of the original data, (i.e., the points in the graphs) are corroborated by the peer-review and publication processes of the journals. The 46 articles processed were published in 17 journals. In December 2021, 15 of them covering 44 articles were indexed by both Web of Science (Science Citation Index Expanded, https://mjl.clarivate.com) and Scopus (https://service.elsevier.com/app/answers/detail/a_id/14834/supporthub/scopus). One article^21^ was published in a journal which was not indexed by any of them, while another article^22^ was published in a journal which was not indexed by Scopus, but was indexed by Web of Science (Emerging Sources Citation Index). Before using the data, however, users of the database should review the original publications, whether the materials and methods used to generate the original data meets the requirements of the usage they plan.

Regarding the most important aspects however, the protocols used for data generation were consistent. The definition of surviving cells was the same in all except one publication^43^. Those cells were considered as survivors, which was able to generate a colony with more than 50 cells after irradiation. While three articles^47,54,60^ do not include this definition of colony formation, the authors of these articles used the same definition in their other publications^49,59,62^. If plating efficiency was mentioned in the article, then it was also stated that surviving fractions after irradiation was calculated considering the plating efficiency of the control i.e., non-irradiated cells. These are in agreement with the protocol of the clonogenic assay described by Franken *et al*.^1^.

On the other hand, differences in the protocols were also found during the review of the Materials and Methods sections. In some cases, the cell cycles of the cells were synchronized, e.g. in^34^, while in other cases they were exposed to hormonal treatment^29^. The time between plating and irradiation also varied cf^23^. and^44^. In addition, cell counting was performed either by hand^49^ or by a computer program^34^.

The technical quality of the collected data was ensured by using two different software for data collection. If there was a larger difference than 0.01 between the numerical values of surviving fractions read by WebPlotDigitizer4.2 and OriginPro2018, the data point was digitized again from the original graphs by both applications. The same quality control procedure was applied to the whiskers. In the case of absorbed doses, it was also considered that the dose values are integer multiples of 0.05 Gy.

While it was a condition for the data to be included in the database that uncertainties of surviving fractions were reported, it is important to note that there is no unique established way of reporting errors in cell survival values^16^. In addition, they still represent only a lower limit concerning the uncertainty of the data and a full uncertainty analysis would be demanding as both stochastic and systematic errors would have to be respected^16^.

In order to ensure the technical quality of the LQ and IR model parameters, a reanalysis was performed by fitting to the digitized data. The LQ model fit converged in all the 101 datasets. The LQ model parameters were provided in the original articles only in 24 cases. From these 24, there was only one dataset^59^ where the parameters obtained by our fit and the parameters of the original article were significantly different.

**Fig. 3:**
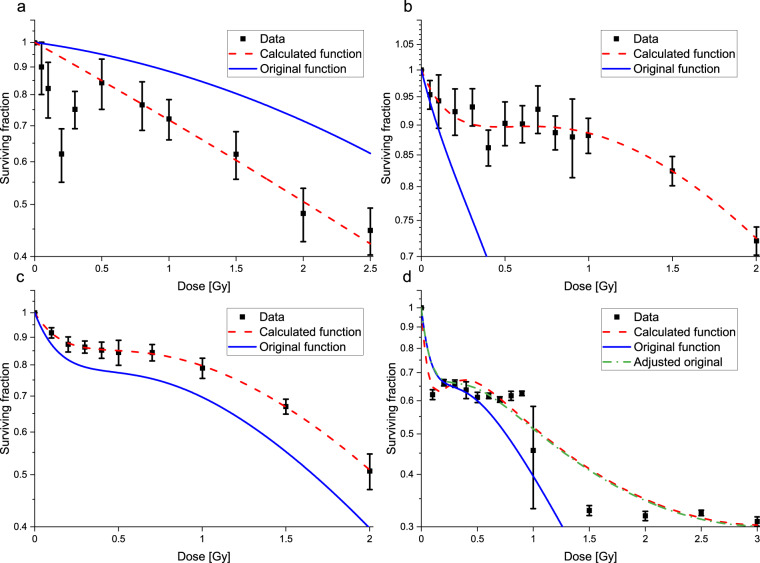
The graphs show the LQ (panel a) and IR (panels b, c, and d) model fits from the original articles, and from the reanalysis we performed. The original models are presented as blue solid lines, while our fit results as red stripped lines. The experimental data are shown as black squares with their uncertainties. The top left panel (**a**)^59^ shows the case where the original LQ fit differed from the one of the reanalysis. The top right (**b**)^33^ and bottom left panel (**c**)^61^ show two IR model cases where we could not reproduce the results of the original fit. In case of the bottom right panel (**d**)^34^, the original fit results could not be reproduced either. However, if the original parameters with a negated *β* value are used, then the curve (green dotted line) fits well to the experimental data.

Our IR model fit did not converge in case of 15 datasets from the total of 101. IR model parameters were not provided in the original articles in case of these 15 datasets. From the remaining 86 datasets where our IR model fit converged, there were 59, where the IR parameters were provided in the original articles. In case of 56 datasets, one of the methods reproduced the original parameters. In case of the remaining three datasets, the original IR parameters could not be reproduced by the fitting procedure we applied. The differences in these three cases can be seen in Fig. 3 as well as in Table 1 (panels and rows b^33^, c^61^, and d^34^).

**Table 1:** Differences between the LQ (row a^59^) and IR (rows b^33^, c^61^, and d^34^) model parameters in the original articles and those obtained by fitting the data read.

		*α* (Mean ± SD)		*β* (Mean ± SD)	
a	Original	0.08*	—	0.044	—
	Calculated	0.32*	± 0.11	0.008	± 0.05
		***α﻿***_***r***_ **(Mean ± SD)**	***β*** **(Mean ± SD)**	***α﻿***_***s***_ **(Mean ± SD)**	***D***_***c***_ **(Mean ± SD)**
b	Original	0.49* ± 0.11	0.52* ± 0.26	1.27 ± 1.18	0.31 ± 0.19
	Calculated	0.005* ± 0.072	0.077* ± 0.036	0.787 ± 0.206	0.333 ± 0.107
c	Original	0.18* ± 0.01	0.14 ± 0.04	1.86* ± 0.05	0.27 ± 0.08
	Calculated	0.043* ± 0.023	0.146 ± 0.013	1.181* ± 0.052	0.296 ± 0.02
d	Original	0.79 ± 0.05	0.13* ± 0.02	5.45 ± 1.35	0.15 ± 0.03
	Calculated	0.783 ± 0.068	−0.128* ± 0.028	9.233 ± 4.191	0.115 ± 0.033

Those parameters are marked with as asterisk (*) where there is a significant difference between the original and our calculated values. In case of the original values of row a, uncertainties were not provided in the article.

## Usage Notes

The database can be used for meta-analysis, model validation, or for comparison with the results of new experiments. Users can download the Microsoft Excel 2016 file. It contains a single sheet with all the 101 datasets. Users can search for radiation type (e.g., ^4^He^2^^+^ or X-rays) or for cell line (e.g., CHO or V79) using the search tool of the application and select relevant datasets for their studies. Datasets can be copied and pasted into other applications where they can be analysed or compared with model predictions or new experimental data. While the database is significantly smaller than the Particle Irradiation Data Ensemble^17^, it may also be useful for the systematic analysis of the datasets included.

## Data Availability

No custom code was used to generate or process the data. WebPlotDigitizer4.2 (GNU Affero General Public License v3.0, https://automeris.io/WebPlotDigitizer/) and OriginPro2018 (OriginLab Corporation, https://www.originlab.com/) were used to obtain numerical values of the data points and their uncertainties plotted in the graphs. The file containing the database was prepared in Microsoft Excel 2016 (Microsoft Corporation, https://www.microsoft.com/en-gb/microsoft-365/excel).
